# Prevalence of cardiovascular risk factors in Spanish HIV-1-infected male inmates

**DOI:** 10.1186/1758-2652-13-S4-P67

**Published:** 2010-11-08

**Authors:** JP Golf, L Sala, J Aramburo, P Sardà, N Perez-Alvarez, JM Llibre

**Affiliations:** 1 Centre Penitenciari Quatre Camins, La Roca del Valles (Barcelona), Spain; 2 Centre Penitenciari Brians II, Barcelona, Spain; 3 Hospital Universitari Germans Trias, Fundació Lluita contra la SIDA, Barcelona, Spain

## Purpose of the study

HIV-1-infected inmates have an increased prevalence of some particular comorbidities. However, the cardiovascular risk(CVR) of this population has rarely been evaluated.

## Methods

Cross-sectional study carried out among 216 male HIV-1 patients in prison. Patients were stratified according to age(<34, 35-39, 40-44, 45-49, 50-54 and >55 years old, respectively)and their CVR was assessed by Framingham(FRAM) equation. The prevalence of some further risk factors was also evaluated: time on antiretroviral therapy, nadir CD4 count, maximum viral load(VL), time on undetectable VL, HCV-coinfection, and cocaine use.

## Results

**Figure 1:**
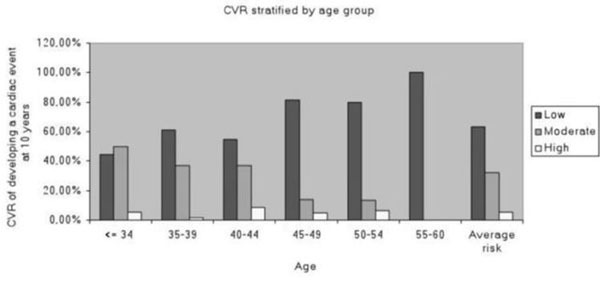
CVR stratified by age group.

Patients median age was 41 years(36-46), their median CD4 count was 386(240- 549)cells, 68% had an undetectable(<50 c/mL)VL, median nadir CD4 count was 207(104-315)cells, and 48% of them had a nadir CD4 count <200 cells. HCV-coinfection prevalence was 94%, cocaine consumption prevalence was 93.1%, and 54.2% of them were intravenous cocaine users. The FRAM 10-years CVR score among subjects studied was 5.88%. Figure 1 and Table 1.

**Table 1:** Cardiovascular risk factors prevalence stratified by age group.

Age	Smokers	Diabetes	Hypertension	Tot Chol > 200	HDL Chol <39
<34	99,44%	0,00%	8,33%	13,89%	72,22%

35-39	100%	3,23%	8,07%	8,07%	43,55%

40-44	100%	3,60%	12,50%	23,20%	51,80%

45-49	100%	0,00%	4,65%	9,31%	60,00%

50-54	100%	26,67%	20%	13,34%	46,00%

55-60	100%	0,00%	75%	100%	0,00%

Overall prevalence	99,07%	3,70%	10,60%	15,27%	53,24%

P	0,455	0,029	0,023	0,001	0,029

Age(p<0.001), total cholesterol(p<0.001), HDL cholesterol(p = 0.029), diabetes mellitus(p = 0.029), hypertension(p = 0.023), and nadir CD4 <200 cells(p = 0.04) were significantly associated with an increased CVR. Smoking, chronic HCV-hepatitis, cocaine use, and the HIV-1 VL were not significantly associated with an increased CVR. There is a trend towards an increased prevalence of hypercholesterolemia and hypertension paralleling the aging.

## Conclusions

Using the FRAM scores, the median CVR of developing a cardiac event at 10 years in a population of Spanish HIV-1-infected inmate males is 5.88%. Of them, 5.1% have a high CVR, and are evenly distributed among age groups. The smoking prevalence is significantly higher than in non-inmate HIV-1 infected individuals, and is so high that it does not allow CVR differences among age groups. HCV-coinfection, cocaine use, and parenteral cocaine consumption were not associated with an increased CVR in our population. On the other hand, a lower nadir CD4 count was associated with high rates of CVR, thus supporting an earlier initiation of ARV therapy in HIV-1 infected males in the prison environment.

